# Interfering with the expression of EEF1D gene enhances the sensitivity of ovarian cancer cells to cisplatin

**DOI:** 10.1186/s12885-022-09699-7

**Published:** 2022-06-08

**Authors:** Qia Xu, Yun Liu, Shenyi Wang, Jing Wang, Liwei Liu, Yin Xu, Yide Qin

**Affiliations:** 1 Department of Biochemistry and Molecular Biology, School of Basic Medical Sciences, Anhui Medical University, 81 Meishan Street, Hefei, Anhui 230032 People’s Republic of China; 2 Department of Obstetrics and Gynecology, the First Affiliated Hospital of Anhui Medical University, Hefei, 230022 Anhui China; 3 Department of Neuropsychology, the Second Affiliated Hospital of Anhui Medical University, Hefei, 230601 Anhui China; 4 Laboratory of Molecular Neuropsychology, School of Mental Health and Psychological Sciences, Anhui Medical University, Hefei, 230032 Anhui China

**Keywords:** Human ovarian cancer, Eukaryotic translation elongation factors 1 δ, Knockdown, Knockout, Xenografted tumor, Gene expression, Signaling pathway

## Abstract

**Background:**

Eukaryotic translation elongation factors 1 δ (EEF1D), has garnered much attention with regards to their role in the drug resistance of cancers. In this paper, we investigated the effects and mechanisms of increasing the sensitivity of ovarian cancer cells to cisplatin or cis-dichlorodiammine platinum (DDP) by knockdown and knockout of EEF1D gene in cellular and animal models.

**Methods:**

The EEF1D gene was knocked-down or -out by siRNA or CRISPR/Cas9 respectively in human ovarian cancer cell SKOV3, DDP-resistant subline SKOV3/DDP, and EEF1D gene in human primary ovarian cancer cell from 5 ovarian cancer patients with progressive disease/stable disease (PD/SD) was transiently knocked down by siRNA interference. The mice model bearing xenografted tumor was established with subcutaneous inoculation of SKOV3/DDP.

**Results:**

The results show that reducing or removing EEF1D gene expression significantly increased the sensitivity of human ovarian cancer cells to DDP in inhibiting viability and inducing apoptosis in vitro and in vivo, and also boosted DDP to inhibit xenografted tumor growth. Interfering with EEF1D gene expression in mice xenografted tumor significantly affected the levels of OPTN, p-Akt, Bcl-2, Bax, cleaved caspase-3 and ERCC1 compared to DDP treated mice alone, and had less effect on PI3K, Akt and caspase-3.

**Conclusions:**

The knocking down or out EEF1D gene expression could enhance the sensitivity of ovarian cancer cells to DDP partially, which may be achieved via inactivating the PI3K/AKT signaling pathway, thus inducing cell apoptosis and decreasing repairment of DNA damage. Our study provides a novel therapeutic strategy for the treatment of ovarian cancer.

**Supplementary Information:**

The online version contains supplementary material available at 10.1186/s12885-022-09699-7.

## Background

Ovarian cancer is the primary cause of mortality in patients with gynecological malignant tumors [1]. The incidence of ovarian cancer has increased significantly, and in recent years the incidence rates have become higher in younger age groups [2]. In total, > 75% of ovarian cancers have been found to have a higher degree of malignancy due to their dormant onset and lack of effective pre-diagnostic methods [3]. Furthermore, ovarian cancer is a malignant cancer that is associated with a high incidence of metastasis and high recurrence after treatment. The current primary method for the effective treatment of ovarian cancer is surgical resection. Chemotherapeutic drugs, such as cisplatin or cis-dichlorodiammine platinum (DDP), carboplatin, and paclitaxel, are the main drugs for patients with advanced ovarian cancer [4–8]. However, a number of patients are faced with tumor recurrence due to tumor dissemination to surrounding organs, which is further complicated with acquired chemoresistance [4, 9, 10]. Over the past few decades, the therapeutic options for the treatment of ovarian cancer have significantly improved through the advancement of surgical techniques and the availability of novel effective drugs that are able to extend the life expectancy of patients [11, 12]. However, due to its complexity and drug resistance, ovarian cancer is still considered one of the most difficult tumors to cure. Therefore, understanding the molecular mechanism of chemoresistance is one of the keys ways to develop novel treatments for ovarian cancer.

The eukaryotic translation elongation factor 1 (EEF1) complex consists of a non-ribosomal proteinase that mediates protein synthesis by recruiting aminoacylated transfer (t) RNA molecules to ribosomes in a GTP-dependent manner [13]. EEF1 is also involved in the recognition of damaged proteins, activation of the proteasomal degradation system and regulation of apoptosis and aging [14, 15]. Recent studies have demonstrated that EEF1 complex proteins also play critical roles in cancer [16, 17]. EEF1 δ (EEF1D), as a guanine nucleotide exchange factor and a subunit of the EEF1 complex, has numerous homotypes [18]. Therefore, EEF1D is not essential for cell survival, and damaging or knocking out the EEF1D gene does not cause cell death [19]. Notably, EEF1D is widely expressed in various human tissues, especially ovarian tissue, see PubMed website (https://www.ncbi.nlm.nih.gov/gene/1936#gene-expression). EEF1D has also been found to be upregulated in numerous malignant tumors, such as liver cancer, esophageal cancer, small cell lung cancer and medulloblastoma [17, 18, 20]. The EEF1D gene located on chromosome 8q24.3 has been found to have several alternatively spliced transcript variants encoding multiple isoforms [19, 21]. The EEF1D gene is conserved in chimpanzees, Rhesus monkeys, dogs, cows, mice, rats, chickens, zebrafish, fruit flies, mosquitos, *Caenorhabditis elegans*, frogs and humans. Therefore, previous studies regarding EEF1D have mainly focused on the relationship between EEF1D and cancers or tumors [22, 23]. EEF1D can activate MAPK and/or PI3K/Akt signaling pathways by activating small G proteins (Ras) to enhance cell repair and anti-apoptotic capacity to increase drug resistance in cancer cells [18, 24]. EEF1D has also been found to promote the expression of heat shock proteins through the activation of heat shock promoter elements, which can inhibit apoptosis induced by multiple chemotherapeutic agents [25]. Therefore, it was hypothesized that knocking down or removing the expression of the EEF1D gene may decrease drug resistance in cancer cells.

In the current study, a loss-of-function EEF1D model in human ovarian cancer cell lines was created and its functions in DDP resistance were evaluated. Furthermore, the signaling pathways altered by EEF1D loss-of-function were explored to investigate the potential underlying mechanisms of EEF1D-induced drug resistance. Overall, this study demonstrated a potential and novel therapeutic target for the treatment of human ovarian cancer.

## Methods

### Reagents

Restriction enzymes (*Bam*HI and *Eco*RI) and T4 ligase were purchased from Sangon Biotech Co., Ltd. Rabbit polyclonal or monoclonal antibodies against human cytokeratin 7 (cat. no. ab181598), Bax (cat. no. ab32503), Bcl-2 (cat. no. ab196495), PI3K (cat. no. ab86714), optineurin (OPTN; cat. no. ab151240), Akt1/2/3 (cat. no. ab184136), phosphorylated (p)-Akt1 (T308; cat. no. ab105731), excision repair cross-complementation group 1 (ERCC1; cat. no. ab129267) and β-actin (cat. no. ab8227) were purchased from Abcam. Rabbit polyclonal antibodies against human caspase-3 p17 (cat. no. sc-271,028) and EEF1D (cat. no. abs136494) were purchased from Santa Cruz Biotechnology, Inc., and Absin Bioscience, Inc., respectively. Lipofectamine® 3000 transfection reagent was purchased from Invitrogen (Thermo Fisher Scientific, Inc.). The HRP-conjugated secondary antibody (goat anti-mouse or goat anti-rabbit IgG, cat. no. PV-6000) were purchased from Beijing Zhongshan Jinqiao Biotechnology Co., Ltd. FITC-labeled goat anti-rabbit IgG (H + L) secondary antibody (cat. no. A0562) and Hoechst 33342 (cat. no. C1022) were purchased from Beyotime Institute of Biotechnology. LY294002 (PI3K inhibitor; cat. no. ab120243) and MK-2206 (Akt inhibitor; cat. no. SF2712) were purchased from Abcam and Beyotime Institute of Biotechnology, respectively.

### Cell cultures

Human ovarian cancer cell line SKOV3 (cat. no. HTB-77), human embryonic kidney cell line 293 (cat. no. CRL-3216) and *Escherichia coli* strain TOP10 (cat. no. PTA-5669) were purchased from the American Type Culture Collection (ATCC). The DDP-resistant human ovarian cancer cell line SKOV3/DDP was purchased from Shanghai Suran Biotechnology Co. Ltd., China. The pLJM1, Δ8.91 and pVSVG plasmids were provided by Wuhan Miaoling Biotechnology Co., Ltd., China. The pLentiCRISPR, pL-SSA-puro, pSPAX2 and pMD2G plasmids were provided by Shanghai GenePharma Co., Ltd. The lentiviral vector systems used in the experiment were all third-generation products. SKOV3, SKOV3/DDP and 293 cells were cultured in DMEM from Gibco with 10% FBS from Gibco in 5% CO_2_ at 37 °C, and then digested with 0.4% trypsin once they reached the logarithmic growth phase for subsequent experiments.

Fresh primary ovarian tumor tissues classified as serous ovarian adenocarcinoma (IV grade) according to the World Health Organization criteria [26] were collected from 5 patients with ovarian cancer with progressive disease/stable disease (PD/SD) who underwent surgery debulking at The First Affiliated Hospital of Anhui Medical University (Hefei, China) between January and December 2019. These patients had received DDP or carboplatin chemotherapy. Prior to tissue deposition, all patients signed written informed consent forms confirming their donation of tissue for research purposes according to the Declaration of Helsinki. All methods were carried out in accordance with relevant guidelines and regulations (eg. Helsinki declaration). This study was approved by Biomedical Ethics Committee of Anhui Medical University (approval no. 20170213; Hefei, China). Fresh primary ovarian tumor tissues were slowly washed with PBS 2–3 times, and the tumor tissue was cut into small pieces (~ 1 mm^3^), transferred to a 50 ml sterile centrifuge tube with 0.4% trypsin and digested at 37 °C for 40–50 min. Then, the tumor tissue fragment was centrifuged at 100 x g for 5 min under 4 °C, and the pellet was resuspended in DMEM with 10% FBS containing 0.1 mg/l EGF, 0.1 mg/l insulin-like growth factor and 0.1 mg/l β-estradiol, then cultured in 5% CO_2_ at 37 °C. After 48 h, the unattached tissues were washed with PBS, and the adherent cells were further cultured until they reached 70–80% confluence, cells were then digested with 0.4% trypsin and subcultured for subsequent experiments. The ovarian cancer cells were identified and analyzed using immunofluorescence staining of cytokeratin 7.

### Knockdown (KD) of EEF1D gene

#### Design and screening of EEF1D small interfering (si)RNA

For EEF1D knockdown (KD), EEF1D siRNA was designed and screened based on the EEF1D gene sequence, and scrambled siRNA served as the control. The sequences were as follows: EEF1D siRNA, 5′-GGUUCGACAAGUUCAAAUATT-3′; and scrambled RNA, 5′-UUCUCCGAACGUGUCACGUTT-3′. EEF1D siRNA and scrambled siRNA were synthesized by Shanghai GenePharma Co., Ltd. EEF1D siRNA or scrambled RNA was transiently transfected into SKOV3 and SKOV3/DDP cells using Lipofectamine® 3000 transfection reagent. According to the open reading frame of EEF1D gene sequence queried from PubMed, PCR primers with low GC content and high specificity were designed and selected with Primer-Blast (Primer 6.0 Software, Canada). The forward (F) and reverse (R) primers of EEF1D were 5′-GTATCTCCCATGCGCCAAGT-3′ and 5′-ATCCAGCAGGATGGAGGACT-3′, respectively. Transfection efficacy was determined via reverse transcription (RT) PCR, with β-actin serving as the control gene.

#### Construction of pLJM1-EEF1D short hairpin (sh) RNA plasmids

The EEF1D shRNA and scrambled shRNA sequences were designed according to the aforementioned siRNA or scrambled RNA in which *Eco*RI and *Bam*HI restriction recognition sites were designed at the end of these sequences (Table S1). The EEF1D shRNA and scrambled shRNA synthesized by Sangon Biotech Co., Ltd., were ligated with the lentiviral vector pLJM1. The pLJM1-EEF1D shRNA and pLJM1-Scrambled shRNA plasmids were transformed into *E. coli* TOP10. The recombinant plasmids were purified, further identified by restriction endonucleases and sequenced.

#### Lentiviral infection and establishment of stably transfected cell lines

The recombinant lentiviral expression vector (pLJM1-EEF1D shRNA and pLJM1-Scrambled shRNA) and the lentiviral packaging plasmid (Δ8.91 and pVSVG) were transfected into 293 cells using Lipofectamine 3000. The detailed protocol was as follows. 293 T cells were seeded into wells of 6-well plates at 2.5 ml (7.5 × 10^4^ cells/ml) /well for culturing in DMEM with 10% FBS at 37 °C for 24 h. The medium was then replaced with serum-free medium, and 3 μg/well pLJM1 and 9 μg/well packaging vectors (Δ8.91 and pVSVG) mixed with Lipofectamine 3000 were added. After culturing for 6 h, the medium containing the transfection mixture was replaced by DMEM containing 10% at 2 ml/well and cell culturing was continued for 72 h. The titers (TU/ml) of virus solution were determined by calculating the number of cells with fluorescence in the maximum dilution multiplication well. After being centrifuged at 4000 rpm at 4 °C and filtered with 0.45 μm syringe filter, the virus stock solution was ultracentrifuged at 20,000 rpm for 2 h, then the supernatant was removed and resuspended in 1 ml of DMEM medium. Last, the viral supernatant was divided into small aliquots and placed at − 80 °C.

SKOV3 and SKOV3/DDP cells were inoculated into a 6-well plate until they reached 70% confluency in 5% CO_2_ at 37 °C. Then, the lentivirus was added to the culture medium to infect the cells with a multiplicity of infection (MOI) of 20 in 5% CO_2_ at 37 °C, meanwhile polybrene was added to the culture medium at a final concentration of 6 μg/ml. The infection was repeated once a day, and its efficiency was observed and identified using a fluorescence microscope with GFP reporter at 72 h post-infection. Using puromycin selection, infected cells were subcultured 4–5 times to obtain a monoclonal cell population, in which EEF1D gene KD was tested via RT-PCR and Western blotting. Each experiment for the electrophoresis of PCR products and Western bloting of proteins was performed in triplicate with two independent sets.

For EEF1D KD in human primary ovarian cancer cell (POCC), the method was the same as aforementioned for SKOV3 and SKOV3/DDP cells, but cells were transiently infected with the lentivirus and infected cells were not selected by puromycin and were not subcultured.

### Knockout (KO) of EEF1D gene

For EEF1D knockout (KO), the single guide RNAs (sgRNAs) were designed according to the specificity and efficiency. The exon 2 region of EEF1D was selected to be targeted via CRISPR/Cas9 genome editing. The target sequence of the gene was 5′-GGCCACGGCCCCACAGACCC-3′, in which the last CC bases were knocked out. The guide (g) RNA of EEF1D was constructed into the vector (site of restriction enzyme *Bsm*BI) to obtain the pLentiCRISPR-EEF1D plasmid. For effective screening of positive cells, SSA vectors were constructed, onto which the target sequence containing the gRNA was constructed to obtain the pL-SSA-puro-EEF1D plasmid. Recombinant lentivirus vectors were produced by transfecting 293 cells with the aforementioned two plasmids and packaging mix plasmids (pSPAX2 and pMD2G) using Lipofectamine 3000, according to the manufacturer’s protocols.

SKOV3/KO and SKOV3/DDP cells in the optimal growth state were infected with the aforementioned lentiviral particles. After the cell culture and selection of single colonies, the genome was extracted from a growing monoclonal cell mass, in which the CRISPR/Cas9 edited site was amplified via PCR and constructed onto a TA vector for sequencing validation. The primers of PCR amplified target sequence were as follows: F, 5′-GGTTGTCCCTAGGACTGTGAG-3′ and R, 5′-GCCCCAGGAAAGACAAAAACT-3′. EEF1D expression in cells was validated via Western blotting. To avoid an off-target effect, potential off-target regions were selected and subjected to PCR and Sanger sequence analysis.

### KD of OPTN gene

For OPTN KD in SKOV3/DDP/KD line, OPTN shRNA and scrambled shRNA sequences were designed in which EcoRI and BamHI restriction recognition sites were designed at the end of these sequences (Table S2). The detailed protocol was the same as aforementioned KD of EEF1D gene. However, cells were transiently infected with the lentivirus and infected cells were not selected by puromycin and were not subcultured.

### Measurement of cell viability and apoptosis

SKOV3, SKOV3/KD, SKOV3/KO, SKOV3/DDP, SKOV3/DDP/KD and SKOV3/DDP/KO cells were seeded into 96-well plates in sextuplicate at a density of 5 × 10^3^ cells/well and incubated with DDP at the following concentrations: 0 (as control), 12.5, 25 and 50 μmol/l for 48 h. Cell viability was measured via a water-soluble tetrazolium 1 (WST-1) assay with a cell viability and cytotoxicity assay kit (Beyotime Institute of Biotechnology), according to the manufacturer’s instructions. The viability of POCC and transfected cells (EEF1D shRNA and scrambled shRNA) was measured using the same aforementioned methods for the other cell lines. In addition, the aforementioned cell lines were also seeded into 96-well plates in sextuplicate at a density of 5 × 10^3^ cells/well, and incubated with the medium containing various drugs (0 as control, 25 μmol/l DDP, 25 μmol/l DDP + 15 μmol/l LY294002 and 25 μmol/l DDP + 10 μmol/l MK-2206) for 48 h. Cell viability was measured according to the aforementioned method. The SKOV3/DDP/KD cells transiently infected with OPTN shRNA and scrambled shRNA were also seeded into 96-well plates in sextuplicate at a density of 5 × 10^3^ cells/well, and incubated with the medium containing various drugs (0 as control and 25 μmol/l DDP) for 48 h, in which cell viabilities were measured according to the aforementioned method. Cell viability was calculated using the following formula: Cell viability ratio (%) = (the experimental group A_450_ nm value/control group A_450_ nm value) × 100%. Each experiment was performed in triplicate independently.

Apoptosis rates of SKOV3, SKOV3/KD, SKOV3/KO, SKOV3/DDP, SKOV3/DDP/KD and SKOV3/DDP/KO cells treated with DDP at 25 μmol/l were measured via flow cytometry (EPICSRXL-MCL; Beckman Coulter, Inc.) using FITC-conjugated Annexin-V and PI from Sigma-Aldrich (Merck KGaA). Saline was used as the control. The experimental operating procedure was performed according to the manufacturer’s protocols. The results of flow cytometry were analyzed using FlowJo v10.6.2 (FlowJo LLC). Cells that stained positive for Annexin-V were counted as apoptotic. The cell apoptosis rates of POCC and cells transiently infected with the lentivirus (EEF1D shRNA and scrambled shRNA) were measured the same as aforementioned cell lines. The experiment was performed in triplicate (cell lines) or duplicate (POCC) in two independent sets.

### Animal xenograft tumor model

#### Animal treatment

A total of 50 healthy female nude mice inbred strain (BALB/cAnN-nu/nu), 14–17 g body weight and 3–4 weeks old, were purchased from Beijing Vital River Laboratory Animal Technology Co., Ltd. (no. 11400700209297). All animal experiments were carried out following the protocol approved by the Institutional Animal Care and Use Committee of Anhui Medical University (approval no. LLSC20170064). All methods and experimental protocols were carried out in accordance with relevant guidelines and following the regulations of the protocols of the Institutional Animal Care and Use Committee. The study was carried out in compliance with the ARRIVE (Animal Research: Reporting of In Vivo Experiments) guidelines. All mice were kept in a specific pathogen-free environment in a sterile room at 22 ± 2 °C, 40–60% relative humidity and a 12 h light/dark cycle with a nutritionally adequate diet and free access to food and drinking water at the Anhui Provincial Center for Medical Experimental Animals. During animal experiments, animal suffering was minimized as much as possible. All nude mice were euthanized at the end of the experiments. All mice were acclimated for 1 week, and then each nude mouse was inoculated subcutaneously in the right armpit with 0.2 ml SKOV3/DDP cell suspension at 1 × 10^7^ cells/ml. After 2 weeks, 24 mice with similar tumor sizes (~ 100 mm^3^) were selected from the aforementioned group of mice, and then randomly divided into four groups (control, DDP, DDP + scrambled shRNA and DDP + EEF1D shRNA) with six mice in each group. The mice were intraperitoneally injected with DDP at 10 μmol/kg body weight once every week for 4 weeks (in the control group, DDP was replaced with saline), meanwhile, DDP + scrambled shRNA and DDP + EEF1D shRNA groups were injected with 100 μl scrambled shRNA lentivirus and EEF1D shRNA lentivirus, respectively, around the tumor tissues once every week. The tumor volumes were measured with Vernier calipers every 5 days and calculated according to the following formula: V = (1/2) ab^2^. V, tumor volume; a, the largest diameter of tumor; b, the minimum diameter of tumor. After 45 days, all nude mice were euthanized according to Guideline for Euthanasia of Laboratory Animal in China (T/CALAS 31–2017, Chinese Society of Experimental Zoology Group Standards). Then xenograft tumors were removed, weighed, collected and frozen in liquid nitrogen for subsequent experiments.

#### Morphological observation of xenograft tumor tissues and TUNEL assay

The tumor specimens were fixed with 4% paraformaldehyde solution at room temperature for 24 h, dehydrated with alcohol, embedded in paraffin and prepared into normal tissue sections. These xenograft tumor tissues were stained with H&E at room temperature for 5 min (H) + 3 s (E) and subsequently observed and analyzed under an optical microscope. The ovarian cancer cell apoptosis of xenograft tumor tissues was analyzed using a TUNEL assay (Roche Diagnostics GmbH), following the manufacturer’s instructions. Apoptosis was quantified using ImageJ software (version 1.44; National Institutes of Health). The apoptosis index (AI) was calculated according to the following formula: AI = (the number apoptotic cells / the total number of cells) × 100%.

### RT-PCR

The aforementioned ovarian cancer cells were harvested, in which the total RNAs were extracted with TRIzol® reagent and RNA extraction buffer according to the manufacturer’s instructions (cat. no. 15596026; Invitrogen; Thermo Fisher Scientific, Inc.). RNA was reverse-transcribed to cDNA using above TRIzol transcription kit following the manufacturer’s instructions. The reverse transcription reaction conditions were 42 °C for 1 h and 70 °C for 5 min. The cDNA amplification in PCR instrument (Veriti 96-Well Thermal Cycler, Thermo Fisher Scientific, Inc.). The reaction conditions were as follows: 94 °C pre-denaturation 5 min; 94 °C × 30 s, 55 °C × 30 s, 72 °C × 45 s (30 cycles). β-actin was used as the housekeeping gene, primer pairs of which (designed by Primer 6.0 and synthesized by Sangon Biotech Co., Ltd.) were used for the PCR: F, 5′-ATCCAGGCTGTGCTATCCCT-3′ and R, 5′-TTGCCAATGGTGATGACCTG-3′.PCR products were observed by 1.5% agarose gel electrophoresis, in which the intensities were semi-quantified using Quantity One software version 29.0 (Bio-Rad Laboratories, Inc.). All reactions were performed in triplicate, and two independent experiments were run.

### Western blotting

Xenograft tumor tissues were lysed in RIPA lysis buffer, and proteins were resolved via SDS-PAGE [5% stacking gel, 10% lower gel (w/v); 22.5 μg protein in 15 μl loaded per lane] and electrotransfered onto PVDF membranes, which were performed using a standard protocol [27]. Protein concentration was measured using a bicinchoninic acid protein assay kit (cat. no. P0012; Beyotime Institute of Biotechnology). Western blotting assays were performed using primary antibodies (1:500 or 1:1000) specific for EEF1D, PI3K, OPTN, Akt, p-Akt, Bcl-2, Bax, caspase-3, cleaved caspase-3, ERCC1 and β-actin. Membranes were then incubated with HRP-conjugated goat anti-mouse or goat anti-rabbit IgG secondary antibodies (1:10,000). The proteins were detected with an ECL system followed by exposure in ChemiScope 6300 Fluorescence and Chemiluminescence Imaging system (Clinx Science Instruments Co., Ltd.), in which digital images were captured and the intensities were semi-quantified using Quantity One software version 29.0 (Bio-Rad Laboratories, Inc.). β-actin was used as the loading control. Each experiment was performed in two independent sets.

### Statistical analysis

All results are presented as the mean ± standard deviation. The differences among groups were analyzed using one-way ANOVA followed by Tukey’s post hoc test with SPSS software (version 20.0; SPSS, Inc.). *P*<0.05 was considered to indicate a statistically significant difference.

## Results

### Culture and identification of human POCCs

**Fig. 1:**
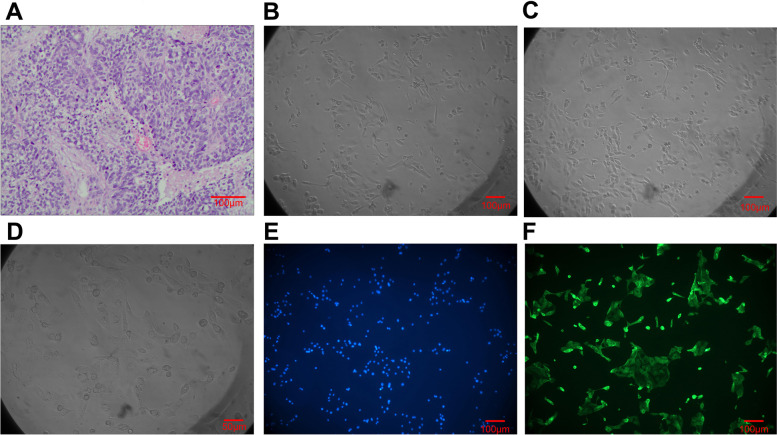
The culture and identification of human primary ovarian cancer cells (× 100, only D × 200). **A** Pathological section of human ovarian cancer tissue from one of the five patients (H&E stained) that was classified as serous ovarian adenocarcinoma (IVgrade) according to WHO criteria. **B, C** and **D **Human primary ovarian cancer cells subcultured in medium from three of the five patients. **E** Cultured human primary ovarian cancer cells stained with nuclear dye, Hoechst 33258. **F** Cultured human primary ovarian cancer cells stained with anti-cytokeratin 7-FITC

POCC lines were successfully isolated and established from 5 patients with ovarian cancer with PD/SD who underwent debulking surgery at The First Affiliated Hospital of Anhui Medical University. These primary cells were cultured in the laboratory and morphologically represented typical cancer cells (Fig. 1 A-D), where Fig. 1 A is a pathological section of H&E stained ovarian cancer tissue in one of the five patients. Figure 1 B, C, and D are human primary ovarian cancer cells subcultured in medium from three of the five patients. Immunocytochemistry analysis of anti-cytokeratin 7 staining revealed an average of ~ 90% ovarian cancer cell purity within the isolated cell populations (Fig. 1 E-F).

### Establishment of stable human ovarian cancer cell lines with EEF1D KD and transient KD of EEF1D gene in human primary ovarian cancer cells

By constructing the pLJM1-shRNA EEF1D plasmid, lentivirus packaging, infecting the target cells and subculturing cells under puromycin screening, the four stable ovarian cancer cell lines (SKOV3/EEF1D shRNA, SKOV3/ scrambled shRNA, SKOV3/DDP/EEF1D shRNA and SKOV3/DDP/scrambled shRNA) were successfully established, among which SKOV3/scrambled shRNA and SKOV3/DDP/scrambled shRNA cell lines were used as the controls. The expression of EEF1D in SKOV3 and SKOV3/DDP cells, in which EEF1D genes were knocked down, were determined via RT-PCR and Western blotting. The expression of EEF1D mRNA in SKOV3/EEF1D shRNA and SKOV3/DDP/EEF1D shRNA cells was significantly decreased compared with the control (Fig. S1). EEF1D protein expression in SKOV3/EEF1D shRNA and SKOV3/DDP/EEF1D shRNA cells was also significantly reduced compared with the control (*P* < 0.01; Fig. S2 A and B). EEF1D protein expression in human POCCs transiently infected with the lentivirus (POCC/EEF1D shRNA) was also significantly decreased compared with the control (*P* < 0.01; Fig. S2 C and D).

### Establishment of stable ovarian cancer cell lines with EEF1D KO

To elucidate the effect of EEF1D in the drug resistance of ovarian cancer, CRISPR/Cas9 was used to KO the EEF1D gene in SKOV3 and SKOV3/DDP cells. sgRNAs were designed to edit the exon 2 region of the EEF1D gene in genomic DNA, thus resulting in gene frameshift mutations (Fig. S3). Through KO and SSA vector construction, lentivirus packaging, infecting the target cells and monoclonal screening, stable ovarian cancer cell lines with EEF1D KO were successfully established, in which the KO of the EEF1D gene was validated by sequencing the target gene and Western blotting in SKOV3 and SKOV3/DDP cells (Fig. S3).

### KD or KO of EEF1D increases the sensitivity of ovarian cancer cells to DDP in vitro

**Fig. 2:**
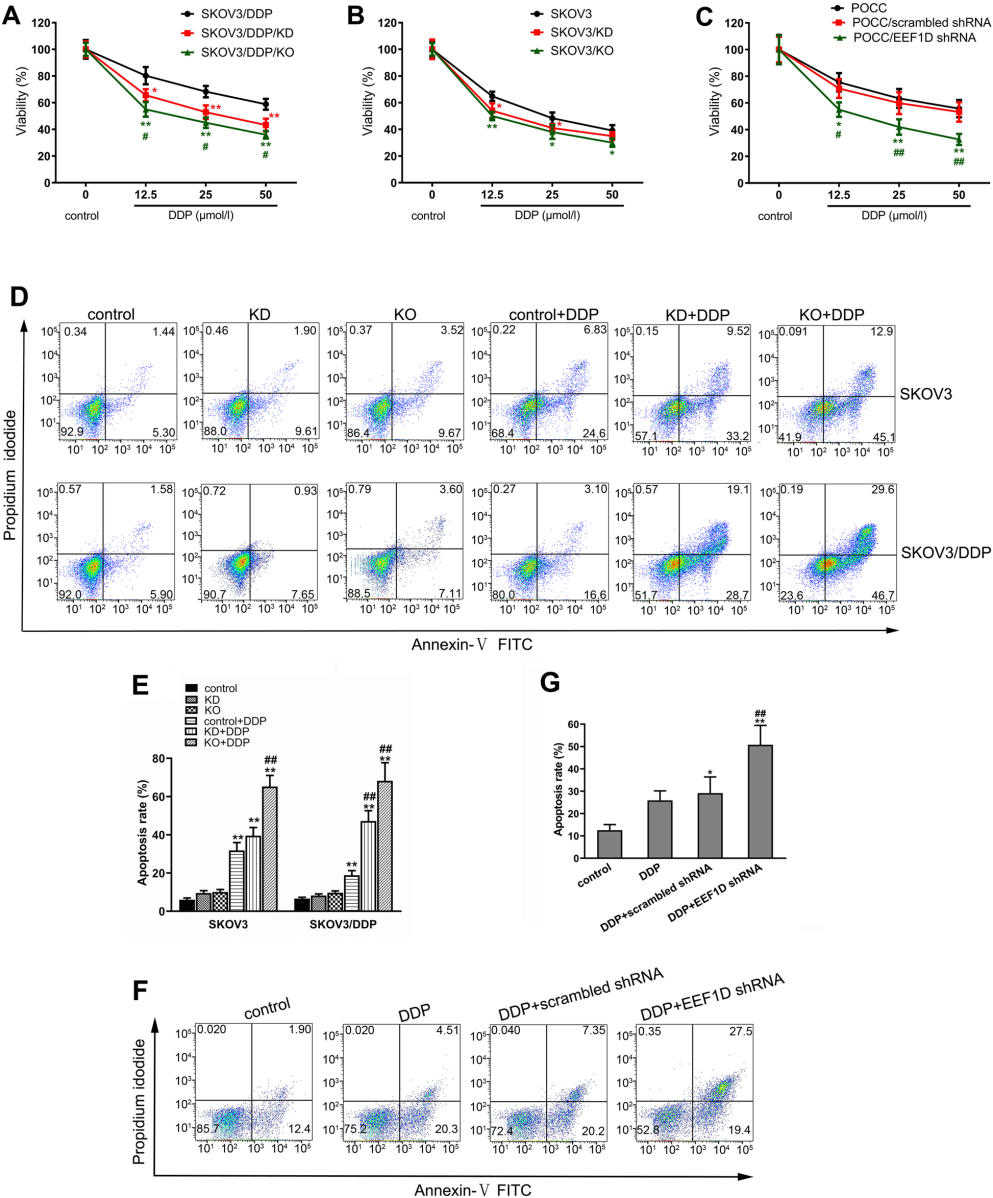
Knocking down or out EEF1D gene expression increased the sensitivity of human ovarian cancer cells to DDP in vitro. **A** The viabilities of ovarian cancer cells with DDP treatment in SKOV3 cell line stably knocked down and out EEF1D gene. **B** The viabilities of ovarian cancer cells with DDP treatment in SKOV3/DDP cell line stably knocked down and out EEF1D gene. **C** The viabilities of ovarian cancer cells with DDP treatment in Human primary ovarian cancer cells transiently knocked down EEF1D gene in 5 ovarian cancer patients. Data in A, B and C are mean ± SD, the experiment was performed with sextuplicate in three independent sets, ^*^P < 0.05, ^**^P < 0.01 compared with SKOV3, SKOV3/DDP or POCC intervened by DDP at the same concentration, ^#^*P* < 0.05, ^##^*P* < 0.01 compared with SKOV3/KD, SKOV3/DDP/KD or POCC/scrambled shRNA intervened by DDP at the same concentration. **D** Representative flow cytometry dot plot of human ovarian cancer cell lines stained with annexin-V-FITC and PI. **E** Histogram of apoptosis rates of human ovarian cancer cell lines. **F** Representative flow cytometry dot plot of human primary ovarian cancer cells stained with annexin-V-FITC and PI in 5 ovarian cancer patients. **G** Histogram of apoptosis rates of POCC. The data in E and G are shown as means ± SD (E:*n* = 6; G:*n* = 10), ^**^P < 0.01 vs. control, KD or KO group, respectively; ^##^*P* < 0.01 vs. control+DDP group. POCC: primary ovarian cancer cell

The ovarian cancer cells were seeded into 96-well plates, grown overnight and treated with different concentrations of DDP for 48 h. Compared with cells without KD or KO of EEF1D expression, the viabilities of ovarian cancer cells with EEF1D KD or KO were significantly decreased following DDP treatment (*P* < 0.05 or *P* < 0.01); the effect in SKOV3/DDP cells was more significant than that in SKOV3 cells (Fig. 2 A and B). Compared with cells with EEF1D KD, the viabilities of ovarian cancer cells with EEF1D KO were significantly decreased following DDP treatment at the same concentration in SKOV3 cells, but there were no significant differences in SKOV3 cells (Fig. 2 A and B). The viabilities of POCCs with transient KD of EEF1D were significantly decreased following DDP treatment (*P* < 0.05 or *P* < 0.01; Fig. 2 C). These results indicated that the ovarian cancer cells were more sensitive to DDP treatment under EEF1D loss-of-function conditions, of which the effect in EEF1D KO cells was more significant than that in EEF1D KD cells.

Using an Annexin V-FITC and PI double-staining method, EEF1D KD and KO were shown to promote the apoptosis of human ovarian cancer cell lines induced by DDP in vitro (*P* < 0.01; Fig. 2 D and E). The effects of KO or KD of EEF1D expression in SKOV3/DDP cells were more significant than those in SKOV3 cells on increasing DDP-induced apoptosis. Also, the apoptosis of POCCs with transient KD of EEF1D expression was significantly increased following DDP treatment (*P* < 0.01; Fig. 2 F and G).

### Reducing the expression of EEF1D gene increases the sensitivity of ovarian cancer to DDP in vivo

**Fig. 3:**
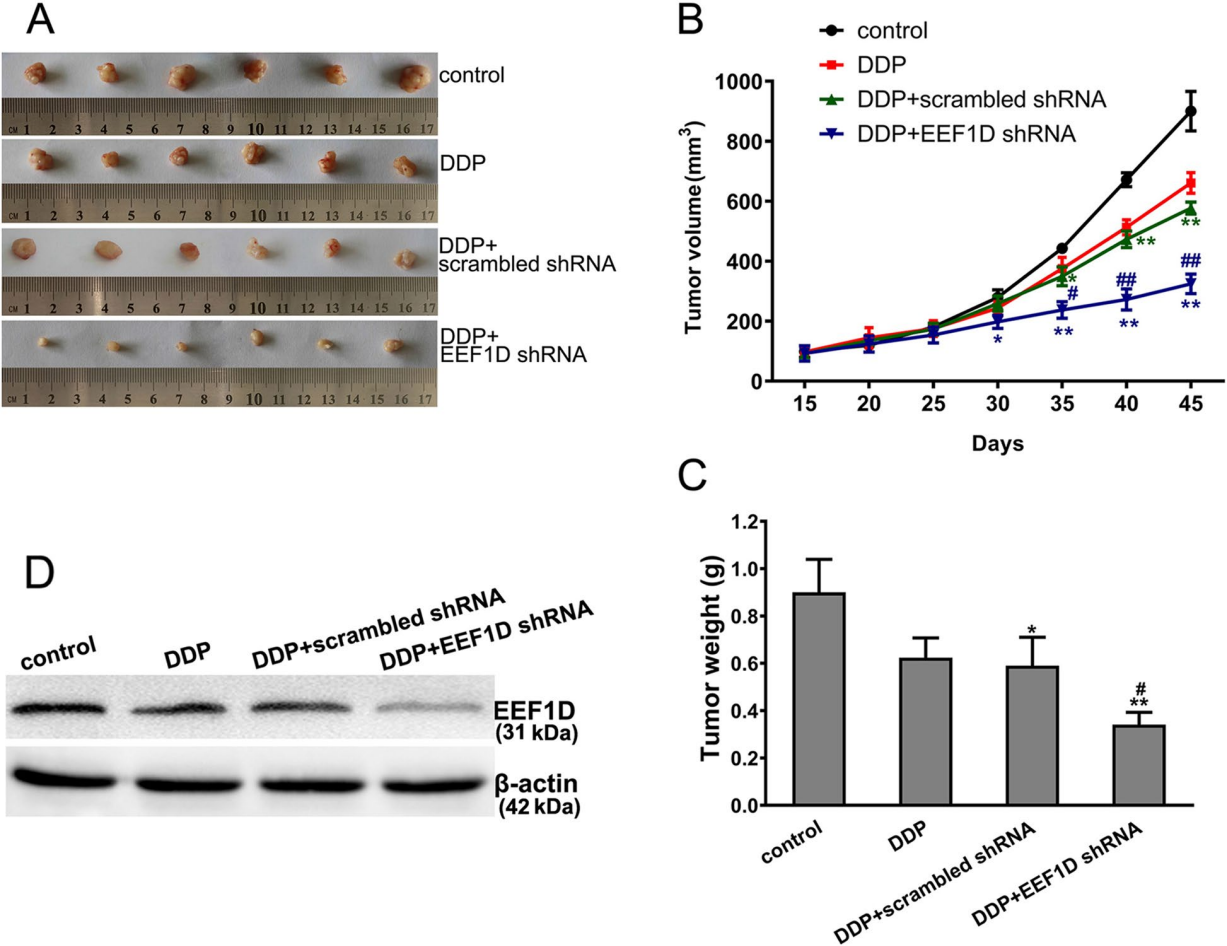
Reducing the expression of EEF1D gene significantly strengthened DDP inhibition of SKOV3/DDP xenografted tumor growth in vivo. **A** SKOV3/DDP Xenografted tumor in tumor-bearing mice. **B** Tumor size. **C** Tumor weight at the end of treatment. **D** EEF1D protein was detected by Western blotting, and β-actin was used to show consistent protein loading. Data are presented as mean ± SD of six mice, ^*^P < 0.05, ^**^P < 0.01 vs. control; ^#^*P* < 0.05, ^##^*P* < 0.01 vs. DDP group

According to our multiple pilot experiments, the SKOV3/DDP EEF1D KO cells do not form tumor in nude mice. So we used SKOV3/DDP EEF1D KD cells for the in vivo study. The results showed that the antitumor effect on SKOV3/DDP cells in the DDP treatment + EEF1D shRNA group was significantly higher than that of the control and DDP treatment alone groups, while the DDP treatment + scrambled RNA group had little effect compared with the DDP treatment alone group (Fig. 3). At the end point, SKOV3/DDP tumors in the control, DDP treatment, DDP treatment + scrambled shRNA and DDP treatment + EEF1D shRNA groups grew to an average volume of 900.23, 660.53, 576.92 and 324.21 mm^3^, respectively (Fig. 3 A and B). Meanwhile, the final tumor weights showed the same trend as tumor volumes (Fig. 3 C). Compared with other groups, the EEF1D expression of the xenograft SKOV3/DDP tumor tissues in DDP treatment + EEF1D shRNA group was significantly reduced with Western blotting test (Fig. 3 D). These results indicated that knocking down EEF1D gene expression could effectively increase the sensitivity of ovarian cancer cells to DDP treatment in vivo. Compared with the DDP group, the tumor growth inhibition rate in the DDP treatment + EEF1D shRNA group was improved by ~ 50%.

### Reducing the expression of EEF1D increases DDP-induced apoptosis in tumor cancer cells

**Fig. 4:**
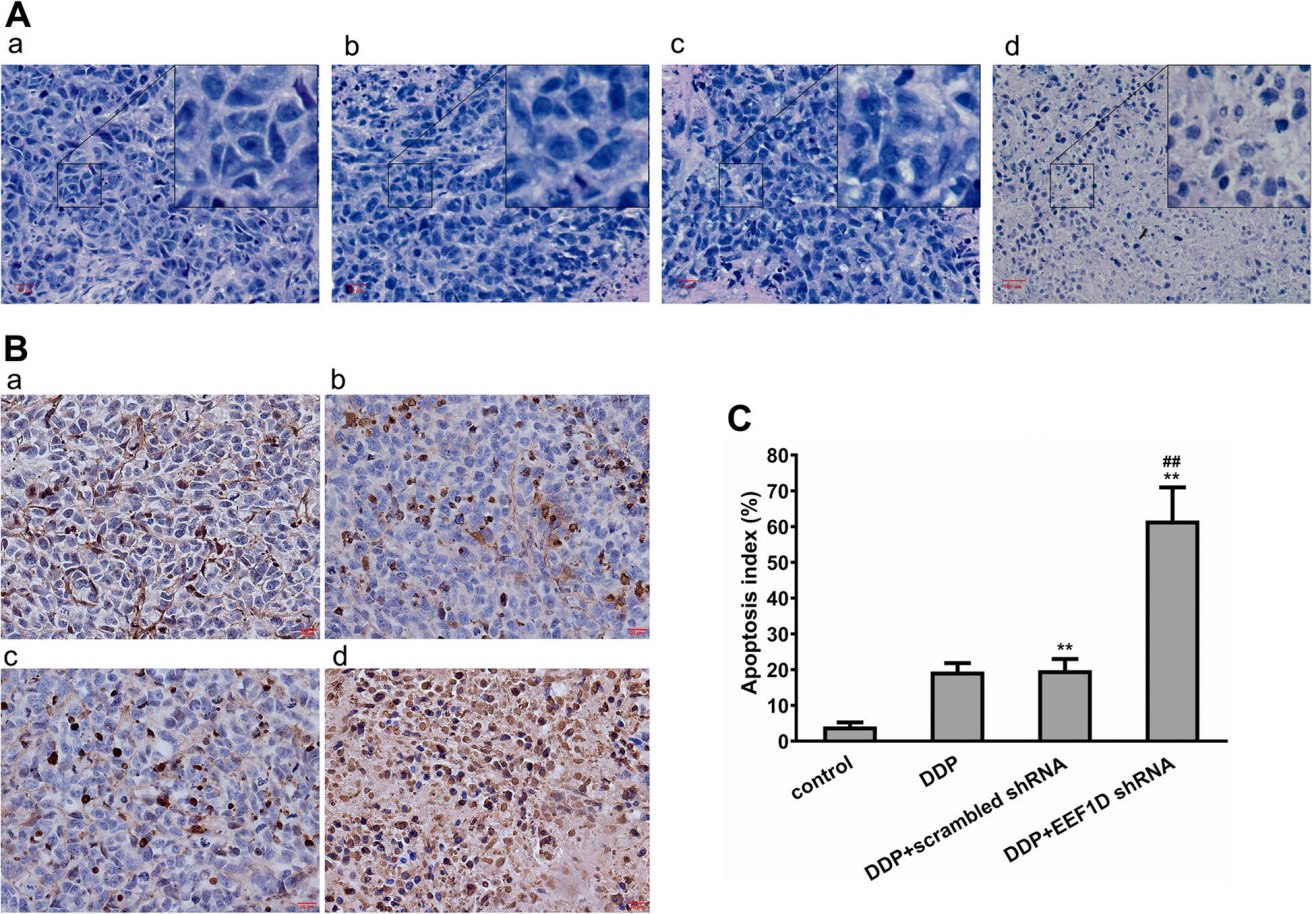
Reducing the expression of EEF1D gene significantly aggravated the pathological changes of SKOV3/DDP xenografted tumor and increased the apoptosis of SKOV3/DDP ovarian cancer cell in xenografted tumor induced by DDP. **A** HE-stained tissue of xenografted tumor; a: control (× 400), b: DDP (× 400), c: DDP + scrambled shRNA (× 400), d: DDP + EEF1D shRNA (× 200). **B** The tumor tissues stained with TUNEL kit (fluorescein-dUTP) and observed under inverted fluorescence microscope (× 400); a: control, b: DDP, c: DDP + scrambled shRNA, d: DDP + EEF1D shRNA. **C** Apoptosis was quantified by Image J software. Data are presented as mean ± SD of six mice in each group. ^**^P < 0.01 vs. control; ^##^*P* < 0.01 vs. DDP group. HE: hematoxylin and eosin

By observing the SKOV3/DDP xenograft tumor tissues stained with H&E under a light microscope, it was found that the control group had intact nuclei and a clear boundary between the nucleus and the cytosol (Fig. 4 A-a), and some cells in the DDP treatment and DDP treatment + scrambled RNA groups showed nuclear condensation, and the ratio of nuclei to cytosol was also increased (Fig. 4 A-b and A-c). However, in the DDP treatment + EEF1D shRNA group, there were highly concentrated nuclei and no cytoplasmic wrap around to form an empty halo, the cells varied in size and the boundaries between cells were not clear (Fig. 4 A-d).

A TUNEL assay was performed on the SKOV3/DDP xenograft tumor tissues in mice. The TUNEL-positive cells of the xenograft tumor tissues in the DDP treatment + EEF1D shRNA group were significantly higher than those in the other groups (*P* < 0.01; Fig. 4 B and C). The apoptosis rates of the control, DDP treatment alone, DDP treatment + scrambled shRNA and DDP treatment + EEF1D shRNA groups were 4.13 ± 1.14, 9.48 ± 2.38, 9.85 ± 3.13 and 61.71 ± 9.26%, respectively.

### Reducing the expression of EEF1D gene affects the levels of proteins associated with cell apoptosis and signaling pathways

**Fig. 5:**
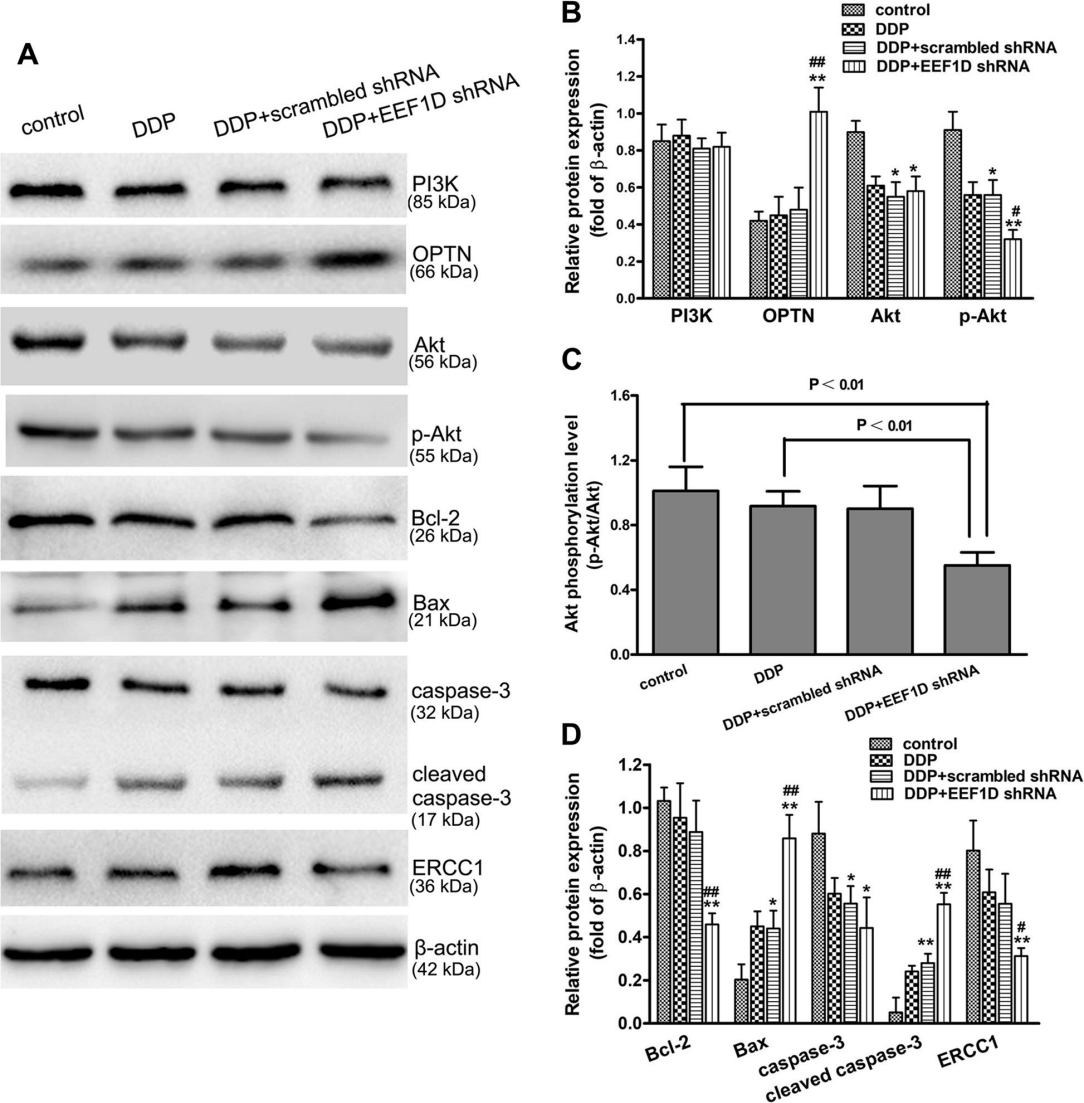
Reducing the expression of EEF1D gene regulated the contents and/or activities of proteins associated with PI3K/Akt signaling pathway, apoptosis and repair of DNA damage in xenografted tumor. **A** The related proteins in xenografted tumor tissues were detected with Western blotting, and β-actin was used to show the similar amount of protein loaded in different lanes. **B**, **D** Relative intensities of protein bands in A were determined using Quantity-One software and normalized using β-actin band intensity. **C** Akt phosphorylation levels were calculated according to the relative intensities of p-Akt/Akt bands, normalized using β-actin. Data in B, C and D are presented as mean ± SD of six mice in each group. ^*^ *P* < 0.05, ^**^ *P* < 0.01 vs. control; ^#^ *P* < 0.05, ^##^ *P* < 0.01 vs. DDP group

Western blotting was performed to analyze the expression levels of PI3K, OPTN, Akt, p-Akt, Bcl-2, Bax, caspase-3, cleaved caspase-3, ERCC1 and β-actin in the xenograft tumor tissues of mice. Though PI3K expression did not notably change in each group, OPTN expression was significantly increased in the DDP + EEF1D shRNA group, thus significantly reducing p-Akt expression (*P* < 0.01; Fig. 5). These results demonstrated that reducing the expression of the EEF1D gene could decrease the phosphorylation of the PI3K/OPTN/Akt signaling pathway. The expression of Bax in the DDP treatment + EEF1D shRNA group was significantly higher than that in the control and DDP treatment alone groups, whereas Bcl-2 expression showed the opposite trend (*P* < 0.01; Fig. 5). The expression levels of caspase-3 and cleaved caspase-3 in the DDP treatment, DDP treatment + scrambled shRNA and DDP treatment + EEF1D shRNA groups were significantly higher than those in the control group, however, their levels in the DDP treatment + EEF1D shRNA group showed the most significant changes, especially for cleaved caspase-3 (*P* < 0.05 or *P* < 0.01; Fig. 5). Meanwhile, the expression of ERCC1 was significantly decreased in the DDP + EEF1D shRNA group compared with the control and DDP groups (*P* < 0.05 or *P* < 0.01; Fig. 5). These results showed that reducing the expression of EEF1D could promote apoptosis and reduce the repair of DNA damage in human ovarian cancer cells.

**Fig. 6:**
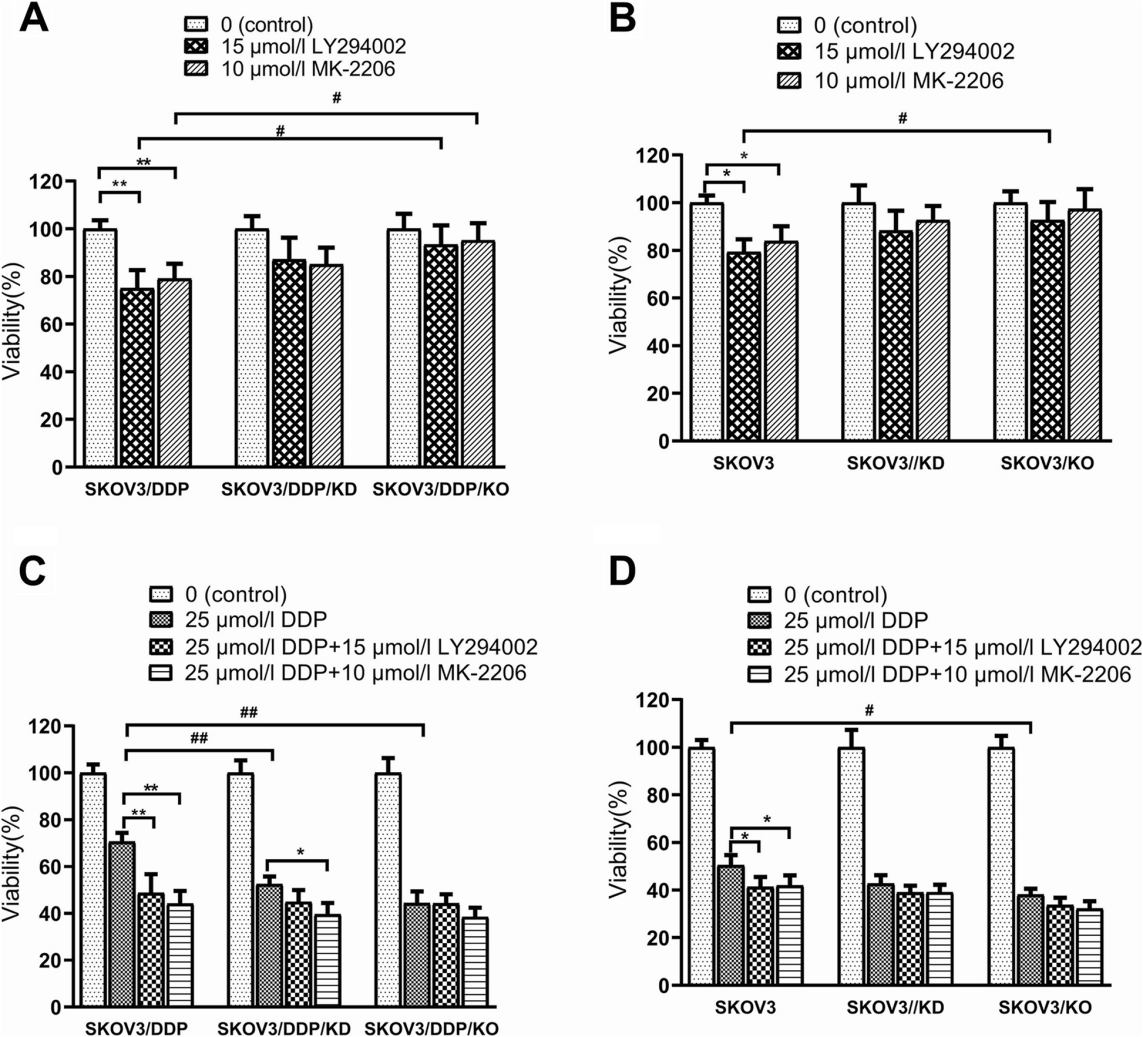
PI3K/Akt inhibitors confirmed that EEF1D could regulate PI3K/Akt signal pathway. **A** and **B** PI3K/Akt inhibitors would decrease viability of EEF1D knockout/knockdown cells less than control cells; **A** SKOV3/DDP cell lines, **B** SKOV3 cell lines. **C** and **D** PI3K/Akt inhibitors had the similar effects from knockout/knockdown cells; **C** SKOV3/DDP cell lines, **D** SKOV3 cell lines. Data are mean ± SD, the experiment was performed with sextuplicate in three independent sets, ^*^P < 0.05, ^**^P < 0.01 compared with DDP intervention alone in the same cell line; ^#^*P* < 0.05, ^##^*P* < 0.01 compared with SKOV3 or SKOV3/DDP in the same drug intervention

**Fig. 7:**
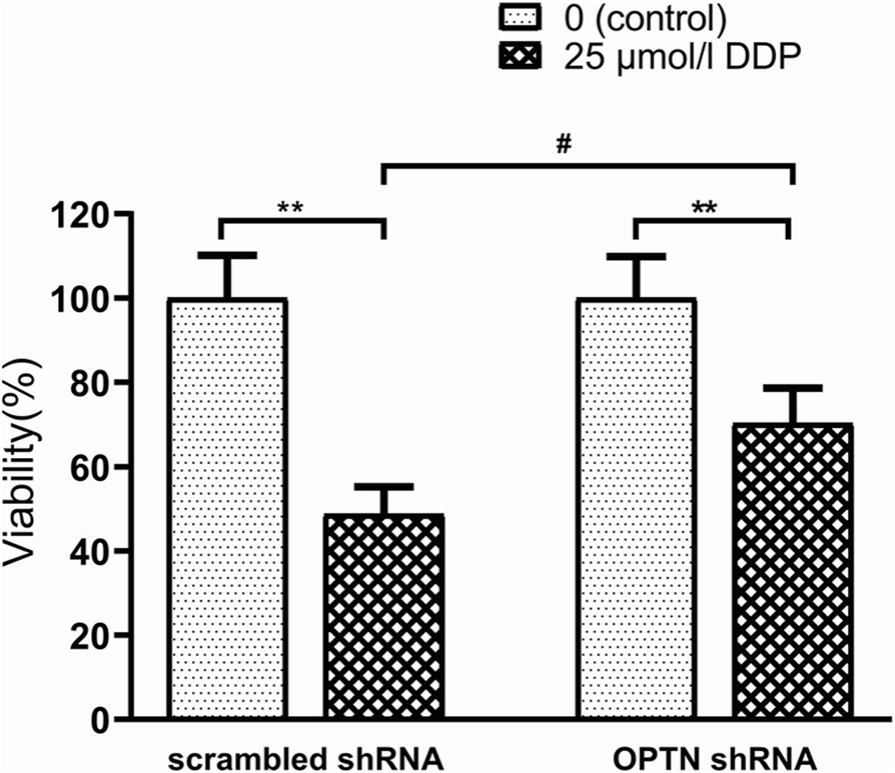
How EEF1D regulates PI3K/OPTN/Akt signal pathway was explored by OPTN knockdown in SKOV3/DDP/KD cell line. Data are mean ± SD, the experiment was performed with sextuplicate in three independent sets, ^**^P < 0.01 vs. scrambled shRNA or OPTN shRNA control, respectively; ^#^*P* < 0.05 vs. scrambled shRNA in the same drug intervention

To investigate the role of the PI3K/Akt pathway, cells were treated with PI3K/Akt pathway inhibitors LY294002 (15 μmol/l) or MK-2206 (10 μmol/l) respectively in the presence or absence of DDP (25 μmol/l). The results demonstrated that PI3K or Akt inhibitors decreased the cell viabilities of EEF1D KO/KD cells less than control cells, they particularly had a low inhibitory effect in KO cells (Fig. 6 A and B). Consistent with the aforementioned results (Fig. 2), EEF1D loss-of-function reduced cell viability following DDP treatment (Fig. 6 C and D, DDP columns). Inhibition of the PI3K/Akt pathway further reduced the viability of SKOV3/DDP (Fig. 6 C) and SKOV3 cells (Fig. 6 D), but had less effect in EEF1D loss-of-function conditions. Since the PI3K/Akt pathway was downregulated in EEF1D loss-of-function conditions (Fig. 5), there was not further potential for the PI3K/Akt inhibitors to suppress this pathway. These results indicated that EEF1D and PI3K/Akt are part of the same pathway in the regulation of the sensitivity of ovarian cancer cells to DDP treatment. To further understand the mechanism of EEF1D affecting human ovarian cancer cell resistance, we performed OPTN knockdown of the DDP-resistant cell line SKOV3/DDP by transient lentiviral infection and found that OPTN KD reversed the effect of EEF1D. Compared with the scrambled shRNA control, OPTN KD can significantly reduce the sensitivity of human ovarian cancer cells to DDP (Fig. 7). These results suggested that decreasing EEF1D expression may regulate the PI3K/OPTN/Akt signaling pathway activity and following cascade (phosphorylation of pro/antiapoptotic proteins) to enhance the sensitivity of ovarian cancer cells to DDP.

## Discussion

The resistance of ovarian cancer cells to chemotherapeutic drugs is the primary cause of tumor chemotherapy failure. The chemotherapy resistance of ovarian cancer is a complex process that involves multiple factors and numerous genes, and there are interactions among the various mechanisms [28]. The mediation of the chemoresistance-associated genes, the hypoxic tumor microenvironment and abnormal regulation of cell apoptosis can cause the ovarian cancer cells to become resistant to anticancer drugs.

The δ of the EEF1 family plays an important regulatory role in the course of cancer, which has been found to be localized in the cytoplasm and membrane of cancer cells. Flores et al [29] found that EEF1D was upregulated in oral squamous cell carcinoma (OSCC) and promoted the activation of cyclin D1 and vimentin, and knocking down EEF1D gene expression could significantly inhibit OSCC proliferation. EEF1D has also been reported to be upregulated in numerous other malignant tumors, such as liver cancer, esophageal cancer, small cell lung cancer and medulloblastoma [17, 18, 20]. EEF1D can activate the PI3K/Akt signaling pathway by activating small G proteins (Ras) to enhance cell repair and anti-apoptotic abilities to increase cancer cell resistance to drugs [24]. EEF1D can inhibit proteasome-mediated apoptosis systems by suppressing ubiquitin ligase, which can inhibit apoptosis induced by a variety of chemotherapeutic agents [23, 30]. Therefore, EEF1D could be considered to be a proto-oncogene when upregulated. Based on its typical and atypical functions, changes in the expression of EEF1D may lead to the transformation and susceptibility of cells to carcinogenesis.

The PI3K/Akt signaling pathway plays an important role in tumor formation, invasion, metastasis and chemotherapeutic resistance, which has been speculated to be a molecular target to reverse chemotherapeutic resistance. When the PI3K/Akt signaling pathway is activated, it affects the ratio of downstream Bax/Bcl-2 activity levels, which is a key determinant of cell susceptibility to apoptosis, rather than individual protein levels [24]. The PI3K/Akt signaling pathway has also been shown to affect the expression of ERCC1, which is responsible for the repair of DNA damage in cells. Jia et al [31] reported that the sensitivity of drug-resistant ovarian cancer cells to platinum was improved by blocking the PI3K/Akt pathway with lysophosphatidic acid receptor gene KD. Previous studies have found that β-elemene could enhance the sensitivity of drug-resistant ovarian cancer cells to DDP, the effect of which may be related to the synergistic induction of apoptosis by β-elemene and DDP [32]. Furthermore, the underlying mechanism may be related to the downregulation of Bcl-2 and survivin gene expression and the upregulation of caspase-3 gene expression.

In this study, we found that EEF1D is a key mediator of chemotherapy resistance to ovarian cancer. After knocking down or knocking out the EEF1D gene, the chemotherapy resistance has been significantly reduced in vitro and in vivo. Mechanistically, we demonstrated that PI3K/OPTN/Akt pathway plays a critical role in EEF1D’s function. This study has two implications. This study firstly demonstrates that EEF1D/PI3K/OPTN/Akt is a novel chemotherapy resistant pathway of ovarian cancer, which partially addresses the molecular mechanism of ovarian cancer chemotherapy-resistance. It is also worth noting that our finding has translational potential. Identifying highly efficient EEF1D’s inhibitors and verifying their chemotherapeutic sensibilization are worth further investigation. Our finding is also consistent with recent studies [18, 33], which have shown that the PI3K/Akt signaling pathway is involved in mediating the regulation of cell resistance.

In the present study, EEF1D expression was successfully reduced or removed in human ovarian cancer cells SKOV3 and SKOV3/DDP using gene KO and KD methods. Although different SKOV3 clones were analyzed, the experiments were performed using a single cell line. For the reproducibility of these data, further research in the future using various cell lines is required. Since the EEF1D KO cell line was compared with its parental cells, the involvement of off-target effects is a possibility, but was not addressed in the current study. However, the consistent results between KO and KD assays indicated that the possibility of off-target effects is very low. It was very difficult to establish stable POCC with KD/KO gene, so POCCs were transiently infected with the lentivirus and were not selected by puromycin to establish stable POCC with KD/KO gene. This study found that although PI3K expression was slightly altered, OPTN significantly increased Akt dephosphorylation, in which p-Akt/Akt was significantly downregulated. The results also showed that the expression of cleaved caspase-3 was significantly increased and ERCC1 was significantly decreased by interfering with the expression of EEF1D. It was suggested that silencing the expression of EEF1D may inactivate the PI3K/Akt signaling pathway and regulate the balance of Bax/Bcl-2 to induce apoptosis, inhibit cell viability and reduce DNA damage repair in ovarian cancer cells. This was partially confirmed by the cell viability assays with PI3K/Akt signaling pathway inhibitors LY294002 and MK-2206, in which PI3K inhibitor (LY294002) and Akt inhibitor (MK-2206) decreased the viabilities of EEF1D KO and KD cells less than control cells. Furthermore, EEF1D overexpression cell lines should be constructed in future experiments to determine whether the overexpression of EEF1D increases Akt activation, and whether the PI3K/Akt inhibitors reverse the effect of EEF1D on increasing the resistance of ovarian cancer cells to cisplatin. OPTN KD experiments showed that reducing OPTN gene expression could abate the effect of EEF1D. We reasoned that EEF1D might be achieved by increasing OPTN and reducing PI3K/Akt pathway function, or through the mutual influence of the two [34–36]. The underlying mechanism of reversing ovarian cancer resistance by silencing the expression of EEF1D needs further experimental confirmation. A significant limitation of this study is the use of subcutaneous injections for the xenograft in vivo experiments. More appropriate orthotopic models will be warranted in future investigations. However, the present study provides novel ideas for elucidating the mechanism of drug resistance in ovarian cancer and provides new avenues to improve the effect of chemotherapy and prolong the survival of patients with cancer.

## Conclusions

We successfully constructed human ovarian cancer cell model knocked down and out EEF1D gene with stable passage, and established an animal model of xenograft mice in human ovarian cancer cell SKOV3/DDP. The reducing or removing EEF1D gene expression could significantly increase the sensitivity of human ovarian cancer cells to DDP both in vitro and in vivo. This may be achieved through inactivating the PI3K/AKT signaling pathway, thus inducing cell apoptosis and decreasing repairment of DNA damage.

## Supplementary Information

**Additional file 1: Figure S1.** Knockdown of EEF1D gene was validated by reverse transcription polymerase chain reaction (RT-PCR). (A) PCR products of EEF1D gene were observed by agarose gel electrophoresis, and β-actin was used as the reference gene. (B) Relative intensity of DNA bands in A was determined using Quantity-One software and normalized using β-actin band intensity. Data shown in B are mean ± *SD* (*n* = 6).

**Additional file 2: Figure S2.** Knockdown of EEF1D gene was validated by Western blotting. (A) EEF1D proteins in SKOV3 and SKOV3/DDP were detected with Western blotting, and β-actin was used to show the similar amount of protein loaded. (B) Relative intensity of protein band in A was determined using Quantity-One software and normalized using β-actin band intensity. (C) EEF1D protein in human primary ovarian cancer cell was detected with Western blotting, and β-actin was used to show the similar amount of protein loaded. (D) Relative intensity of protein band in C was determined using Quantity-One software and normalized using β-actin band intensity. Data shown in B and D are mean ± SD (B: n = 6; D: *n* = 10). POCC: primary ovarian cancer cell.

**Additional file 3: Figure S3.** Knockout of EEF1D gene was validated by DNA sequencing and Western blotting. (A) Genomic sequence of EEF1D exon 2 region targeted by sgRNA editing. (B) Knockout of EEF1D gene in monoclonal cell mass was validated by sequencing. (C) EEF1D protein was determined by Western blotting.

**Additional file 4: Table S1.** The DNA sequences of EEF1D shRNA and scrambled shRNA.

**Additional file 5: Table S2.** The DNA sequences of OPTN shRNA and scrambled shRNA.

## Data Availability

The datasets used and/or analyzed during the current study are available from the corresponding author on reasonable request.
