# Supplementary figures and images for: Interfering with the expression of EEF1D gene enhances the sensitivity of ovarian cancer cells to cisplatin
Source: BMC Cancer. 2022 Jun 8;22:628. doi: 10.1186/s12885-022-09699-7 (PMC9175347; doi:10.1186/s12885-022-09699-7)

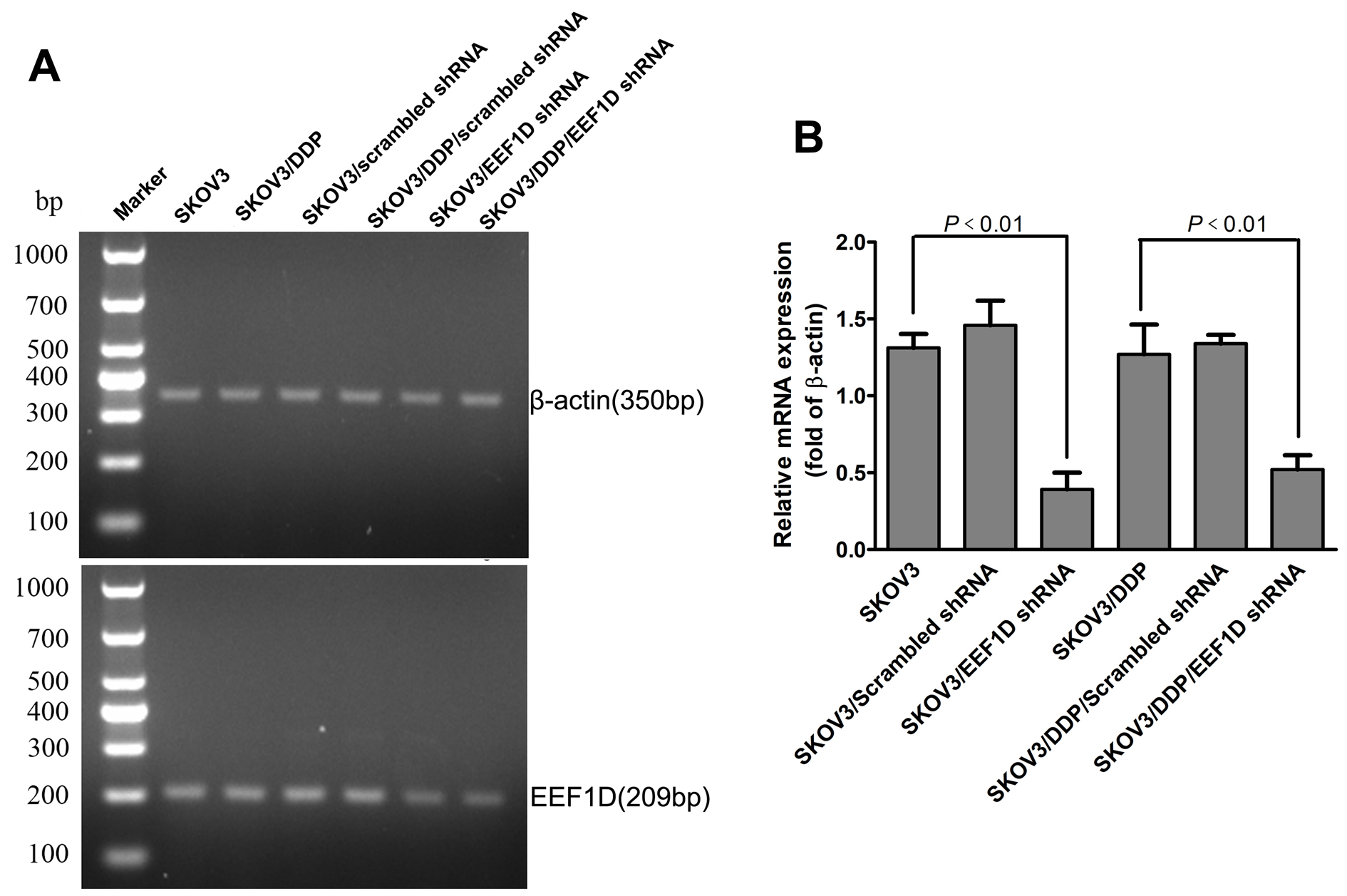

Supplement: Supplementary file 1 — Additional file 1: Figure S1. Knockdown of EEF1D gene was validated by reverse transcription polymerase chain reaction (RT-PCR). (A) PCR products of EEF1D gene were observed by agarose gel electrophoresis, and β-actin was used as the reference gene. (B) Relative intensity of DNA bands in A was determined using Quantity-One software and normalized using β-actin band intensity. Data shown in B are mean ± SD (n = 6). [file 12885_2022_9699_MOESM1_ESM.jpg]

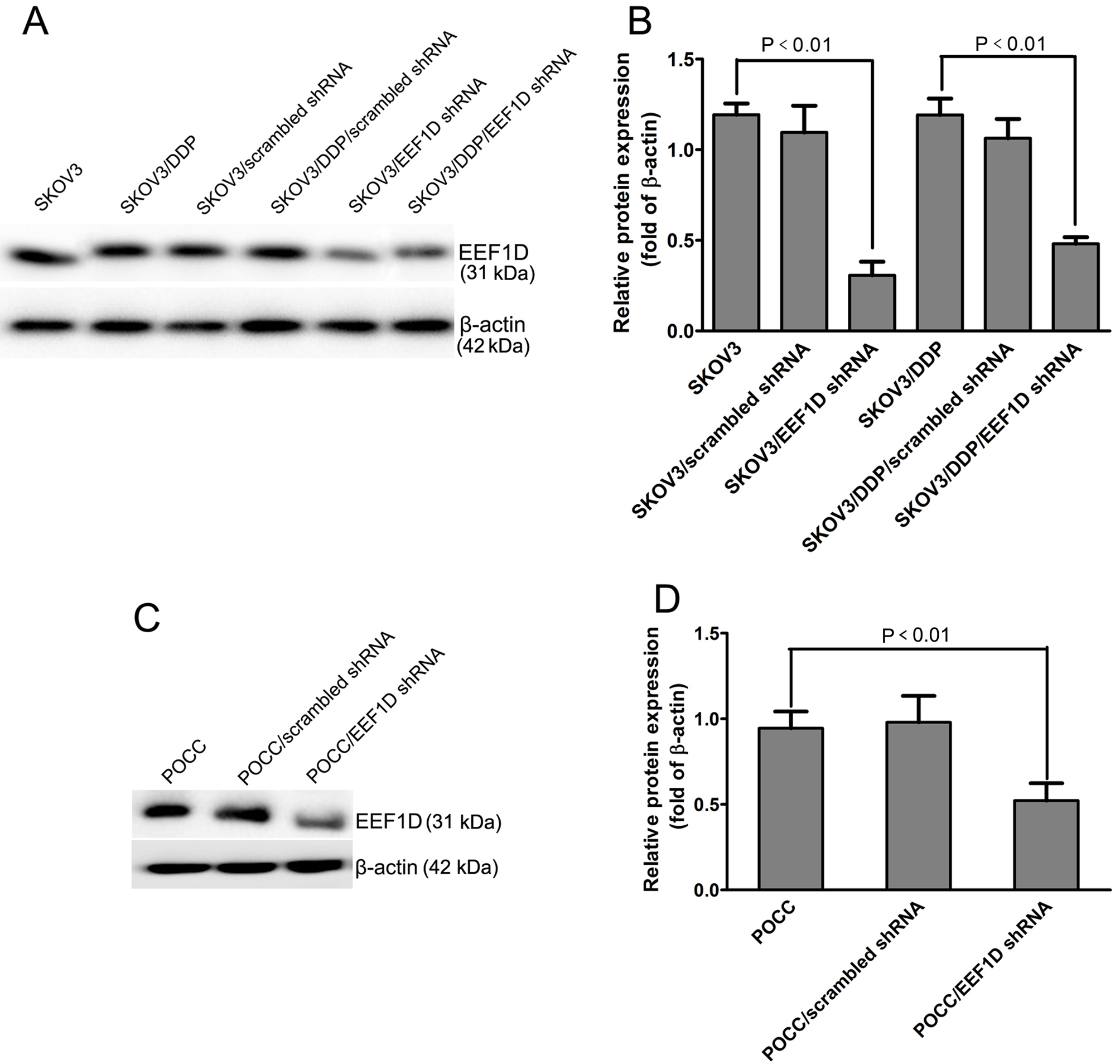

Supplement: Supplementary file 2 — Additional file 2: Figure S2. Knockdown of EEF1D gene was validated by Western blotting. (A) EEF1D proteins in SKOV3 and SKOV3/DDP were detected with Western blotting, and β-actin was used to show the similar amount of protein loaded. (B) Relative intensity of protein band in A was determined using Quantity-One software and normalized using β-actin band intensity. (C) EEF1D protein in human primary ovarian cancer cell was detected with Western blotting, and β-actin was used to show the similar amount of protein loaded. (D) Relative intensity of protein band in C was determined using Quantity-One software and normalized using β-actin band intensity. Data shown in B and D are mean ± SD (B: n = 6; D: n = 10). POCC: primary ovarian cancer cell. [file 12885_2022_9699_MOESM2_ESM.jpg]

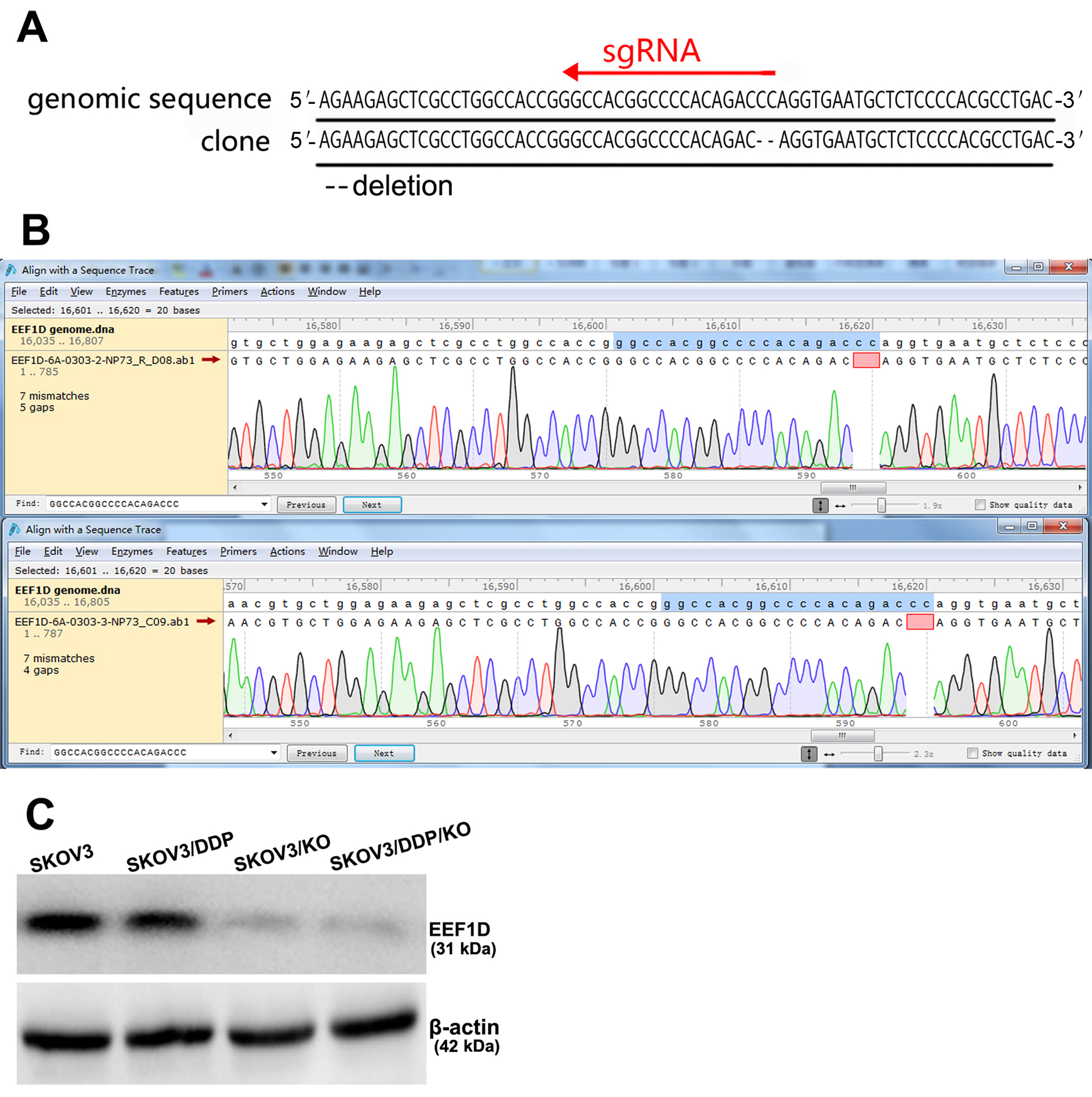

Supplement: Supplementary file 3 — Additional file 3: Figure S3. Knockout of EEF1D gene was validated by DNA sequencing and Western blotting. (A) Genomic sequence of EEF1D exon 2 region targeted by sgRNA editing. (B) Knockout of EEF1D gene in monoclonal cell mass was validated by sequencing. (C) EEF1D protein was determined by Western blotting. [file 12885_2022_9699_MOESM3_ESM.jpg]
