# Supplementary material for: Interfering with the expression of EEF1D gene enhances the sensitivity of ovarian cancer cells to cisplatin
Source: BMC Cancer. 2022 Jun 8;22:628. doi: 10.1186/s12885-022-09699-7 (PMC9175347; doi:10.1186/s12885-022-09699-7)
Supplement: Supplementary file 5 — Additional file 5: Table S2. The DNA sequences of OPTN shRNA and scrambled shRNA. [file 12885_2022_9699_MOESM5_ESM.docx]

**Table S2. The DNA sequences of OPTN shRNA and scrambled shRNA**

|  | DNA sequence |
| --- | --- |
|  |  |
| EEF1D shRNA | F:5'-CACCGTTCTCCGAACGTGTCACGTTTCAAGAGAACGTGACACGTTCGGAGAATTTTTTG-3'  R:5'-GATCCAAAAAATTCTCCGAACGTGTCACGTTCTCTTGAAACGTGACACGTTCGGAGAAC-3' |
|  |  |
| scrambled shRNA | F:5'-CACCGGAGACTGTTGGAAGCGAAGTTTCAAGAGAACTTCGCTTCCAACAGTCTCCTTTTTTG-3'  R:5'-GATCCAAAAAAGGAGACTGTTGGAAGCGAAGTTCTCTTGAAACTTCGCTTCCAACAGTCTCC-3' |
